# Elucidating the effects of organic vs. conventional cropping practice and rhizobia inoculation on rhizosphere microbial diversity and yield of peanut

**DOI:** 10.1186/s40793-023-00517-6

**Published:** 2023-07-18

**Authors:** Dev Paudel, Liping Wang, Ravin Poudel, Janam P Acharya, Samantha Victores, Cleber Henrique Lopes de Souza, Esteban Rios, Jianping Wang

**Affiliations:** 1 Agronomy Department, University of Florida, Gainesville, FL USA; 2 Department of Plant Pathology, University of Florida, Gainesville, FL USA

**Keywords:** Microbiome, Rhizosphere, 16S rRNA, Soil, Peanut, Nodules, Legume, Organic, Inorganic, Conventional

## Abstract

Legumes such as peanut (*Arachis hypogea*) can fulfill most of their nitrogen requirement by symbiotic association with nitrogen-fixing bacteria, rhizobia. Nutrient availability is largely determined by microbial diversity and activity in the rhizosphere that influences plant health, nutrition, and crop yield, as well as soil quality and soil fertility. However, our understanding of the complex effects of microbial diversity and rhizobia inoculation on crop yields of different peanut cultivars under organic versus conventional farming systems is extremely limited. In this research, we studied the impacts of conventional vs. organic cultivation practices and inoculation with commercial vs. single strain inoculum on peanut yield and soil microbial diversity of five peanut cultivars. The experiment was set up in the field following a split-split-plot design. Our results from the 16 S microbiome sequencing showed considerable variations of microbial composition between the cultivation types and inoculum, indicating a preferential association of microbes to peanut roots with various inoculum and cropping system. Alpha diversity indices (chao1, Shannon diversity, and Simpson index) of soil microbiome were generally higher in plots with organic than conventional inorganic practices. The cultivation type and inoculum explained significant differences among bacterial communities. Taxonomic classification revealed two phyla, TM6 and Firmicutes were significantly represented in inorganic as compared to organic soil, where significant phyla were Armatimonadetes, Gemmatimonadetes, Nitrospirae, Proteobacteria, Verrucomicrobia, and WS3. Yields in the organic cultivation system decreased by 10–93% of the yields in the inorganic cultivation system. Cultivar G06 and T511 consistently showed relative high yields in both organic and inorganic trials. Our results show significant two-way interactions between cultivation type and genotype for most of the trait data collected. Therefore, it is critical for farmers to choose varieties based on their cultivation practices. Our results showed that bacterial structure was more uniform in organic fields and microbial diversity in legumes was reduced in inorganic fields. This research provided guides for farmers and scientists to improve peanut yield while promoting microbial diversity and increasing sustainability.

**Supplementary Information:**

The online version contains supplementary material available at 10.1186/s40793-023-00517-6.

## Introduction

Nitrogen is an important nutrient required for plant production, but it is often the most limiting plant nutrient in agricultural systems [1]. Applying large-scale agriculture supplements that supply nitrogen, including nitrates, urea, and ammonium formulation, is not a sustainable practice [2]. Therefore, environment friendly alternatives like organic farming and biological nitrogen fixation (BNF) are getting increasing attention of scientists and farmers [1]. Legume-rhizobia symbiosis can alleviate the need of manufactured nitrogen for farming systems by providing biologically fixed nitrogen to supply plant nutrients and is fundamental to sustainable agriculture [3]. Meanwhile, organic crop growers, who use compost, manure, organic fertilizers, and cover crops for fertilization [4], help to build and improve soil fertility in terms of physical, chemical, biological, and microbiological diversity [5]. Compared to conventional farming methods, organic farming results in higher soil organic matter, microorganism abundance, species richness, and have lower soil erosion, nitrogen loss, and pesticide leaching [6]. However, organic nutrients alone are not enough to increase crop yields to meet global food demand [7] and they perform particularly poorly for vegetables and some cereal crops like wheat [8]. Legumes and perennials show better performance in organic systems because they are more efficient in using nitrogen [8]. The production gap between organic and conventional agriculture increases in the absence of synthetic nitrogen fertilizers [9]. In addition, organic agriculture also has a significantly lower temporal stability (-15%) compared to conventional agriculture [10]. Nevertheless, organic farming enhances soil microbial activity [11], which in return increases productivity of biological nitrogen fixation and can help decrease abundance of harmful pathogens and increase abundance of beneficial bacteria [12, 13]. An increased microbial community sustains and improves soil quality, health, and productivity, further promoting sustainable agriculture.

As legumes, peanuts can supply their own nitrogen by symbiotic association with root nodulating bacteria called *Bradyrhizobia*. However, this symbiosis supplies only 55% of nitrogen needed for peanut’s optimal growth [14]. Due to this, conventional peanuts are often supplemented with chemical fertilizers as an additional source of nitrogen. In addition to nitrogen, various factors can significantly impact peanut yield, including cultivar, agronomic practice, nutrient availability, and soil quality [15]. Therefore, a comprehensive evaluation of these major factors on peanut yield with focus on sustainable agriculture is necessary to get a clear picture of how these factors influence crop yield and the ecosystem. The availability of nutrients to legume crops, including peanuts, is influenced by the plant, rhizobia inoculant and other microbiomes, environmental conditions, and their interactions [16, 17]. Much of these interactions occur in the rhizosphere, which is inhabited by diverse population of microbes [18]. The activities of these microbes significantly influences crop health, nutrition, soil structure, fertility, and yield [19]. The composition of these microbes is driven by the host genotype producing unique root exudates in rhizosphere and agricultural practices such as fertilization, tillage, and type of cropping system [20, 21]. Long term organic system is associated with improved soil structure and function and increased microbial diversity and population [5, 22].

There is a dearth of literature on the complex interactions of cultivars, farming practice, and inoculum and how they impact peanut yield and the soil microbiome. This limited knowledge restricts our ability to fully exploit peanut nitrogen-fixing capacity and sustainable farming practices to improve peanut yield. Filling this gap could increase our understanding of how the interaction between peanut cultivars, rhizobium inoculum, and farming practices could be optimized to increase peanut yield and the sustainability of ecosystem. A relatively high nitrogen-fixation efficient strain of *Bradyrhizobium* (strain Lb8) has been isolated from peanut nodules, and its genome was sequenced and assembled [23]. This strain was used as one of the sources of inoculum in this study. The objective of this research is to determine the effects of organic vs. inorganic farming and different rhizobia inoculums on the yield and soil microbial community of five peanut cultivars. The findings from this project will help farmers using different practices to identify peanut varieties that produce superior yield while improving soil microbial diversity and to understand the effects of rhizobium inoculation on peanut yield under different farming conditions.

## Results

### Diversity of soil microbial community

#### Alpha diversity index

**Fig. 1:**
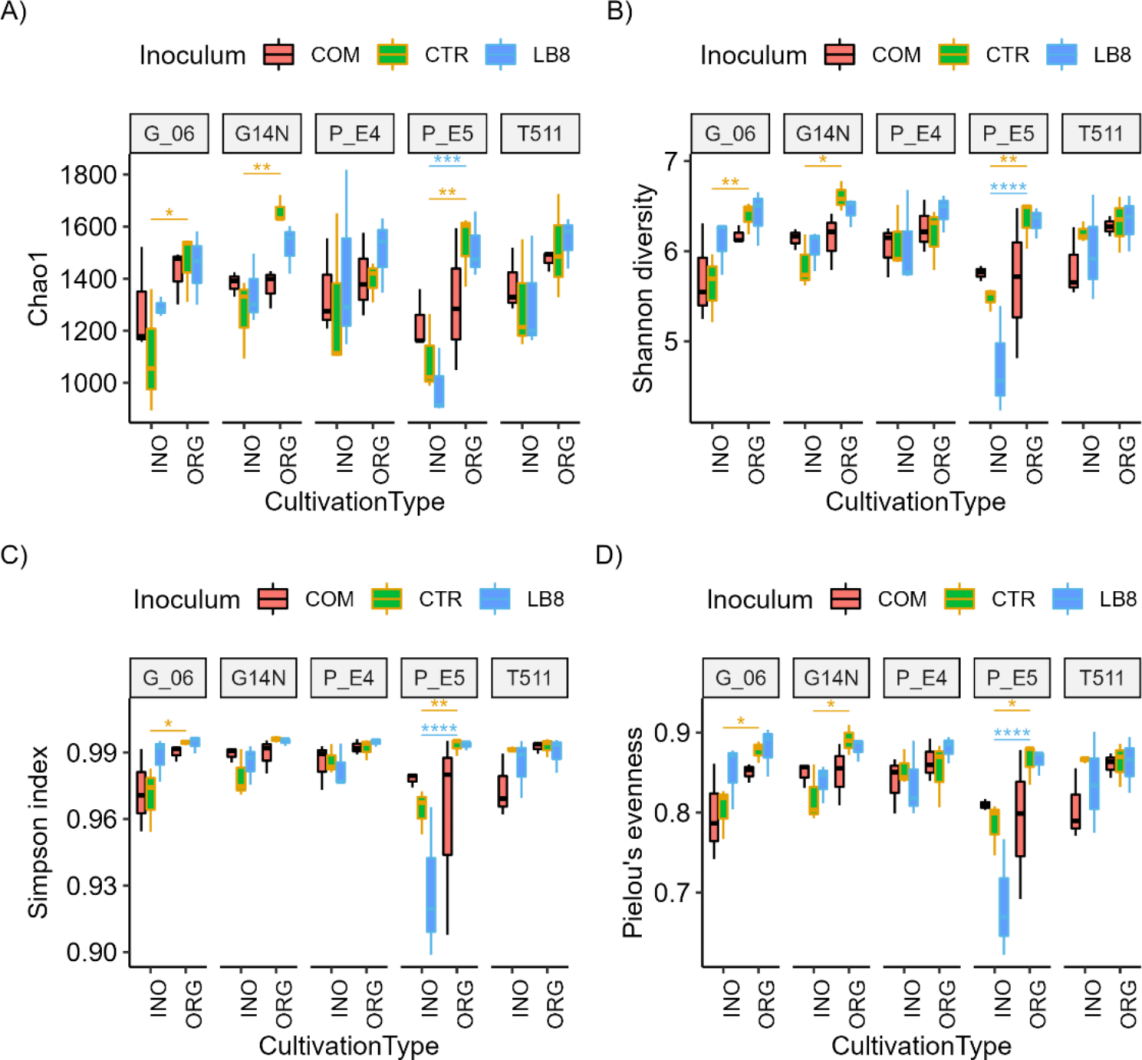
Alpha diversity indices (chao1, Simpson, and Shannon) and Pielou’s evenness for different genotypes in the organic and inorganic plots for different inoculums. Cultivation type (INO = inorganic, ORG = organic); Inoculum (COM = commercial, CTR = control, LB8 = LB8) are separated by fill color; 5 genotypes are grouped separately. Bar above boxplots show significant differences between cultivation types (*p < 0.05; ** *p* < 0.01; **** *p* < 0.0001) and the bar color represents inoculum

The microbial community’s Chao1 diversity was significantly affected by interaction between genotype and cultivation type (Supplementary Table 1A). In the inoculum control group, G06, G14N, and E5 in organic fields had significantly higher soil microbial diversity than those in inorganic fields (Fig. 1A). For LB8 inoculum, only genotype E5 in organic plots had significantly higher (*p* < 0.05) microbial chao1 diversity than that in inorganic plots. Three-way interactions between the cultivation practices, inoculum, and genotype significantly affected both Shannon’s diversity and Simpson index of soil microbiome (Supplementary Table 1B and Supplementary Table 1 C). Shannon diversity was significantly higher (*p* < 0.05) in organic fields compared to inorganic field for LB8 inoculated and uninoculated genotype E5 (Fig. 1B). Similarly, Shannon index was significantly higher (*p* < 0.05) in organic field for uninoculated genotypes, G06, G14N, and E5 (Fig. 1B). The Simpson index was also significantly higher (*p* < 0.05) in organic fields compared to inorganic fields for genotype E5 inoculated by Lb8 and uninoculated. Simpson index was also significantly higher (*p* < 0.05) in organic fields for uninoculated G06. Three-way interactions significantly (*p* < 0.05) affected Pielou’s evenness of soil microbiome (Supplementary Table 1D). Pielou’s evenness was significantly greater in organic fields as compared to inorganic fields for Lb8 inoculated genotype E5. Similarly, Pielou’s evenness was significantly higher in organic fields (*p* < 0.05) of uninoculated G06, G14N and E5. Interestingly, for E4 and T511, the microbial community’s Chao1 diversity, Shannon index, Simpson index, and Pielou’s evenness were the same regardless of whether organic or inorganic practices or inoculum sources were used. These results suggested that microbiome diversity of different peanut genotypes responded differently to farming system and inoculation. In comparison between the sister inbred lines, non-nodulating (Nod-) E4 and nodulating (Nod+) E5 in responding to all the treatments, it showed that the diversity of microbial community in Nod + E5 plots was generally decreased in the inorganic plots, which, however, was not the case for Nod- E4. There were no big differences of microbial diversity between organic and inorganic plots for Nod- E4. This result suggested that inorganic practice could reduce microbial diversity of legume crops with nodulation capacity, and the normal legume plant nodulation or symbiosis process may impact the soil microbial diversity.

### Bacterial community structure

**Fig. 2:**
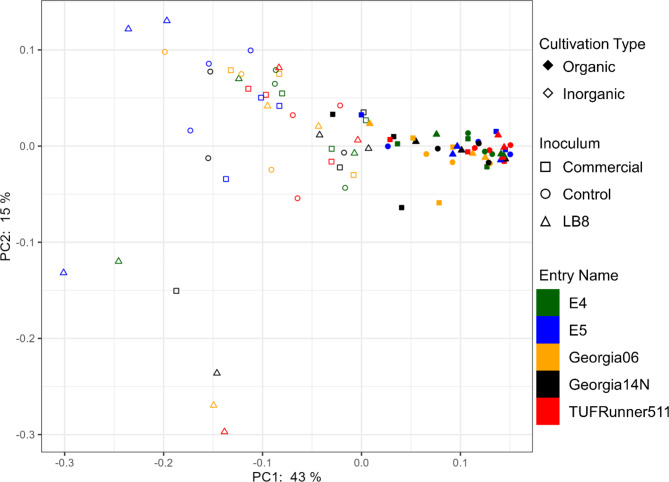
PCoA plot of bacterial community generated by using the weighted UniFrac as distance. Cultivation type is represented by fill (organic = filled, inorganic = unfilled). Inoculum is represented by shape (commercial = square, control = circle, triangle = LB8). Entry names are represented by different color of each shape (E4 = darkgreen, E5 = blue, Georgia06 = orange, Georgia14N = black, and TUFRunner511 = red)

Beta diversity analysis based on Bray-Curtis distance matrix showed that the interaction between cultivation type and inoculum had a significant impact on bacterial community structure. Visualization of these distances using weighted uniFrac as distance showed that the first two principal components were able to explain 58% of the total variation of the bacterial community (Fig. 2). Samples of organic practice no matter the different inoculum source or cultivars were clustered together while the sample of inorganic practice were scattered widely. The results suggested that the bacterial structure was more similar and uniform in organic fields and bacterial structure varied in inorganic fields and influenced by inoculum source more than by cultivars (Fig. 2).

**Fig. 3:**
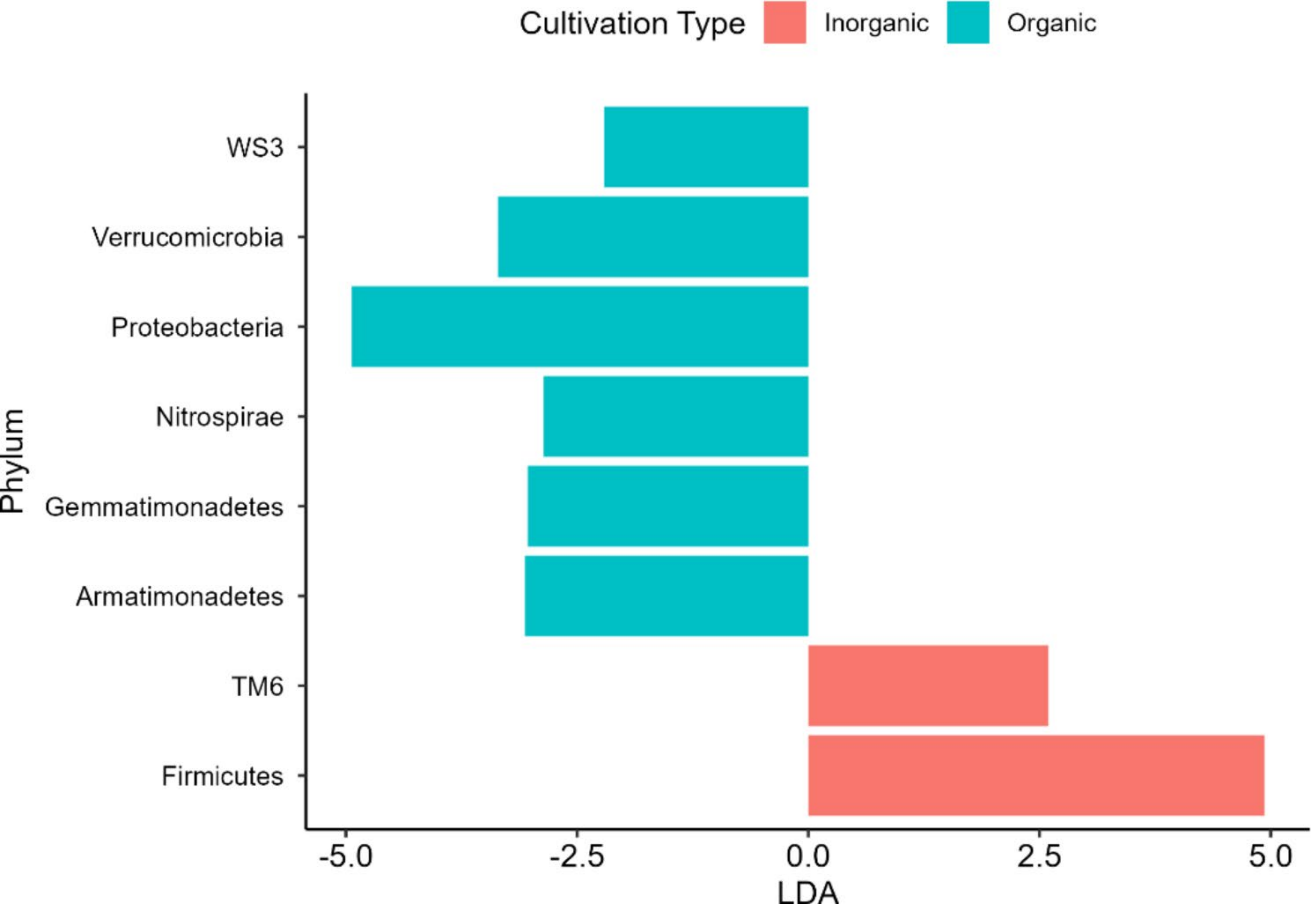
Linear Discriminant Effect Size (LefSe) analysis of bacterial features showing Operational Taxonomic Units (OTU) abundance for different cultivation types at the phylum level. X-axis shows the linear discriminant analysis (LDA) effect size and y-axis shows different phylums

The linear discriminant analysis effect size (LefSe) was used to identify bacterial features represented between organic and inorganic cultivation practices. LefSe analysis confirmed two features (TM6 and Firmicutes) (Fig. 3), showing statistically significant and biologically consistent differences in inorganic soils as compared to organic soil. In the organic soil, six Operational Taxonomic Units (OTUs) were statistically significant including Armatimonadetes, Gemmatimonadetes, Nitrospirae, Proteobacteria, Verrucomicrobia, and WS3. Similarly, at the family level, LefSe analysis confirmed 88 OTUs statistically significant in organic cultivation (Supplementary Figure S1) including Rhizobium, Ralstonia, Burkholderia while OTUs including Acidopila, Lactobacillus, and Bacillus were significant in plots of inorganic cultivation practice.

### Taxonomy barplots

**Fig. 4:**
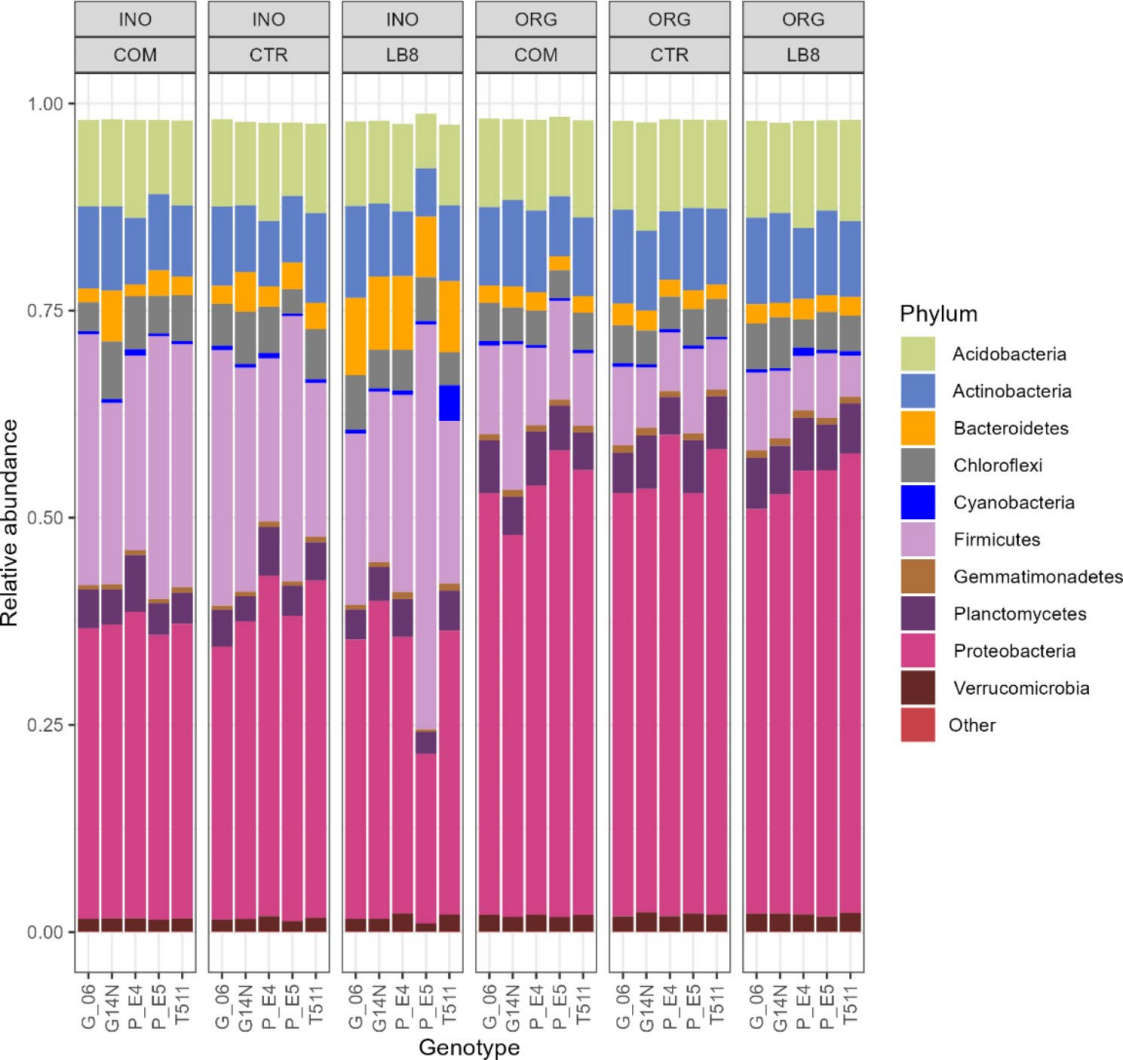
Phylum composition of peanut rhizosphere

**Fig. 5:**
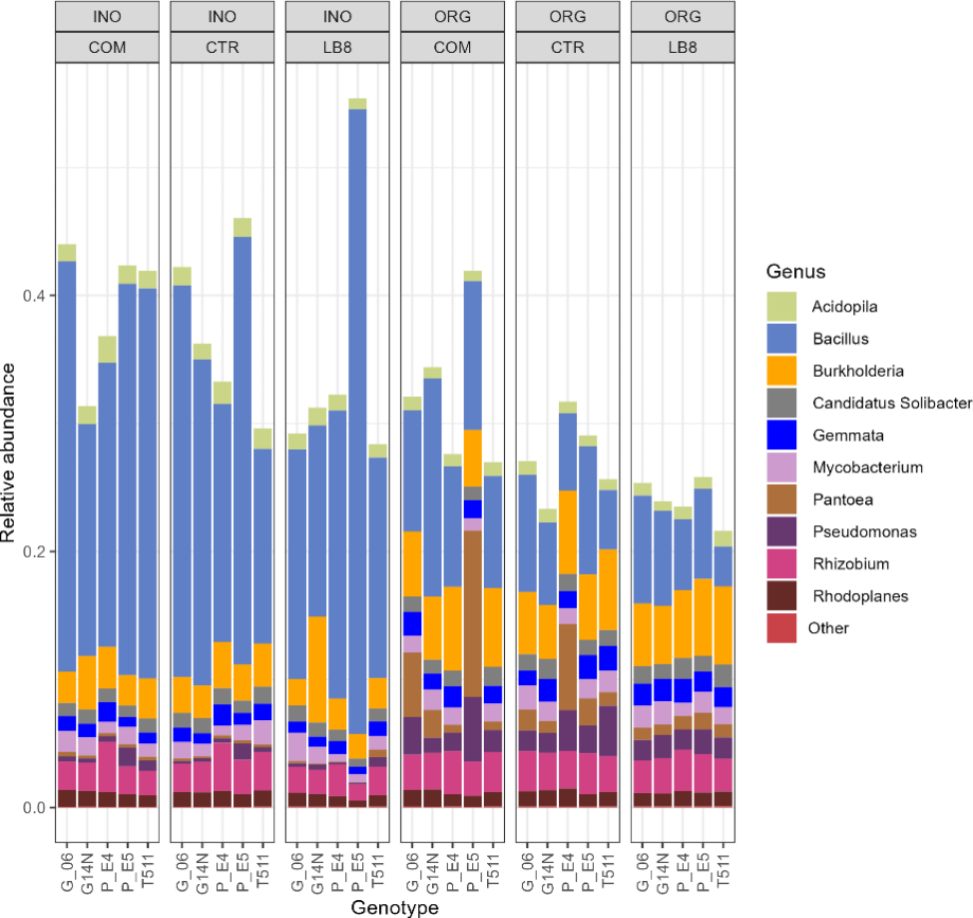
Genus composition of peanut rhizosphere

The top 10 phyla that dominated soil bacterial communities were Proteobacteria, Firmicutes, Bacteroidetes, Actinobacteria, Acidobacteria, Cyanobacteria, Chloroflexi, Planctomycetes, Verrucomicrobia, and Gemmatimonadetes (Fig. 4). At the genus level, the following genus dominated soil bacterial communities: *Bacillus*, *Burkholderia*, *Rhizobium*, *Pantoea*, *Pseudomonas*, *Mycobacterium*, *Gemmata*, *Candidatus Solibacter*, *Acidopila*, *Rhodoplanes* (Fig. 5). The relative abundance of proteobacteria significantly increased while Firmicutes decreased in the organic fields (Fig. 4), and this was largely attributed to species from the genus Bacillus (Fig. 5). Interestingly, in the inorganic plots, the Nod+ E5 had lower abundance of proteobacteria and higher abundance of Firmicutes than its Nod- sister line E4. This difference was not distinct in the organic plots. This result suggested that inorganic practice could reduce the abundance of proteobacteria and enhance the abundance of Firmicutes for nodulating legume crops, while organic practice could alleviate the different impacts on bacterial community abundance between Nod+ and Nod- plants.

### Yield related parameters

Summary of yield parameters evaluated is provided in Table 1. The interaction between cultivation type and genotype had significant impacts on pod yield (Supplementary Table S2A), shoot mass (Supplementary Table S2B), pod number (Supplementary Table S2C), nodule number (Supplementary Table S2D), room mass (Supplementary Table S2E), and SPAD1 and SPAD2 (Supplementary Table S2F, Supplementary Table S2G), but not SPAD3 (Supplementary Table S2H), while there was no significant impact of inoculum on all the above yield related traits. Since E4 is Nod- and is incapable of fixing atmospheric nitrogen into plant soluble nitrogen, it showed the lowest value for all the yield related traits mentioned above, even though organic and inorganic fertilizers were applied to the field (Figures S2). Particularly, the E4 yield related traits were significantly lower in organic than in inorganic plots, while the yield related traits of its sister Nod+ line, E5 were all comparable between organic and inorganic plots. The results indicated that nodulation or symbiosis capacity is an important contributing feature for legume crop yield in organic field to compete that in inorganic field.

**Table 1:** Summary of yield related parameters in organic and inorganic plots treated with three inoculums (COM = Commercial, CTR = Control, and LB8) for five genotypes (G14N = Georgia14N, G_06 = Georgia06, P_E4 = E4, P_E5 = E5, T511 = TUFRunner 511)

Cultivation	Inoculum	Genotype	Yield (Kg/ha)	Shoot Mass (gm)	Pod Number	Root Mass (gm)	Nodule Number	SPAD1	SPAD2	SPAD3
**Organic**	**COM**	**G14N**	2600 ± 155	52.1 ± 11	39.9 ± 1	2.56 ± 0.29	192 ± 42	41.3 ± 1.76	44.7 ± 1.2	33.2 ± 2.17
		**G_06**	3910 ± 527	64.6 ± 6.92	50.8 ± 6.85	3.67 ± 0.193	272 ± 50.4	43.3 ± 0.333	48.7 ± 1.45	41.4 ± 2.33
		**P_E4**	104 ± 28	6.3 ± 0.577	3.1 ± 0.9	1.44 ± 0.294	0 ± 0	19 ± 2	17.3 ± 0.667	14.2 ± 2.91
		**P_E5**	1990 ± 718	70.6 ± 9.84	20.2 ± 5.82	3.78 ± 0.485	228 ± 3.49	39 ± 1.15	41.7 ± 1.45	31.3 ± 3.48
		**T511**	3720 ± 295	48.1 ± 7.43	45.9 ± 4.03	2.11 ± 0.11	195 ± 15.5	40.7 ± 0.333	44.7 ± 1.86	30.1 ± 3.31
	**CTR**	**G14N**	3680 ± 811	56.9 ± 7.79	55.9 ± 8.74	3.11 ± 0.402	187 ± 18.6	41 ± 1	49 ± 1.15	31.8 ± 2.62
		**G_06**	4990 ± 597	59.3 ± 3.9	59 ± 6.96	2.89 ± 0.402	216 ± 43.7	45.3 ± 2.73	56.3 ± 0.882	40.9 ± 0.606
		**P_E4**	104 ± 8	6.33 ± 0.689	2.63 ± 0.333	1.55 ± 0.223	0 ± 0	19.3 ± 1.45	15.7 ± 1.33	13.2 ± 0.769
		**P_E5**	2130 ± 575	83.2 ± 10.6	24.8 ± 5.73	3 ± 0.577	263 ± 53	39.7 ± 1.45	45 ± 1.53	30.3 ± 4.78
		**T511**	2770 ± 298	31.4 ± 3.1	30.2 ± 2.26	2.22 ± 0.223	139 ± 8.94	41.7 ± 0.667	46.3 ± 1.45	28.8 ± 2.64
	**LB8**	**G14N**	4510 ± 715	76.3 ± 9.87	71.3 ± 11.9	3.89 ± 0.402	254 ± 46.6	43.7 ± 1.2	51.3 ± 1.86	31.9 ± 1.28
		**G_06**	3210 ± 561	50.7 ± 15.1	36.1 ± 6.76	3.56 ± 0.588	181 ± 64.7	46 ± 1	51.3 ± 1.76	39.8 ± 1.13
		**P_E4**	168 ± 23	6 ± 0.681	4.43 ± 0.133	1.11 ± 0.22	0 ± 0	17.3 ± 0.882	18 ± 1.15	12.3 ± 2.8
		**P_E5**	2770 ± 583	76.7 ± 14.4	31.9 ± 7.05	3.44 ± 0.728	205 ± 52.2	40 ± 0.577	42.7 ± 1.33	32.6 ± 2.35
		**T511**	1860 ± 269	25.2 ± 2.69	21.2 ± 2.11	2 ± 0.51	157 ± 53.5	40.7 ± 1.2	44.7 ± 0.667	28 ± 3.13
**Inorganic**	**COM**	**G14N**	4360 ± 1200	85.6 ± 7.43	64 ± 10.9	4.44 ± 0.113	347 ± 59.9	39.3 ± 2.73	48.7 ± 2.03	36.6 ± 0.524
		**G_06**	10,600 ± 3200	141 ± 19	133 ± 33	5.55 ± 0.223	392 ± 24.8	42.7 ± 0.667	48.7 ± 1.45	38 ± 0.854
		**P_E4**	1050 ± 566	58.9 ± 22.4	24.5 ± 10.5	2.89 ± 0.78	0 ± 0	31 ± 3.06	26.3 ± 1.2	18.5 ± 2.37
		**P_E5**	2960 ± 650	67.8 ± 16.8	34.9 ± 5.95	3.56 ± 0.483	271 ± 45.5	44.7 ± 1.2	46 ± 0.577	36.6 ± 2.25
		**T511**	7710 ± 1770	67.8 ± 18.1	81.5 ± 21	2.89 ± 0.485	251 ± 58.2	38.7 ± 1.45	46.3 ± 1.67	31.5 ± 1.46
	**CTR**	**G14N**	5720 ± 937	83.8 ± 14	78.2 ± 6.31	3.78 ± 0.619	279 ± 45.3	43 ± 1.15	48 ± 1	37 ± 0.353
		**G_06**	9820 ± 1560	90.9 ± 7.45	113 ± 13.2	3.33 ± 0.193	364 ± 62.4	46 ± 1	50 ± 2	39.6 ± 2.57
		**P_E4**	662 ± 375	33.6 ± 14.4	18.1 ± 9.62	2.33 ± 0.577	0 ± 0	28.3 ± 3.71	24.7 ± 1.2	18.2 ± 1.42
		**P_E5**	1830 ± 227	55 ± 13.4	22.7 ± 1.66	3.11 ± 0.802	264 ± 15.3	44 ± 0.577	46 ± 0.577	29.6 ± 2.91
		**T511**	7260 ± 3380	55.6 ± 22.9	75.3 ± 34.1	2.22 ± 0.779	244 ± 72.9	41.7 ± 0.333	43.7 ± 2.6	29.4 ± 2.15
	**LB8**	**G14N**	5150 ± 872	79.2 ± 7.45	71.9 ± 10.4	4.11 ± 0.588	284 ± 34.3	41.7 ± 1.45	48.7 ± 0.882	32.9 ± 1.72
		**G_06**	6110 ± 1100	92.1 ± 8.19	80.1 ± 13.8	4.78 ± 0.485	352 ± 19	44.3 ± 0.667	50.3 ± 2.03	37.1 ± 1.32
		**P_E4**	1110 ± 235	40.4 ± 5.44	26.5 ± 6.02	2.89 ± 0.22	0 ± 0	27.7 ± 1.2	26.7 ± 1.76	16.4 ± 2.95
		**P_E5**	2980 ± 394	94.9 ± 9.36	36.7 ± 3.47	4.22 ± 0.675	261 ± 34.3	42.7 ± 0.333	46.3 ± 0.667	32.9 ± 1.33
		**T511**	6110 ± 364	67.9 ± 15	78.5 ± 6.95	2.78 ± 0.294	279 ± 38.4	40 ± 1.15	47 ± 2.08	30.3 ± 3.08

Values are means ± SEM, n = 3 per treatment group

#### Pod yield

In the organic plots, yield of G06 was significantly higher (*p <* 0.05) than E4 and at par with rest of the four genotypes (Supplementary Fig. 2A). Similarly, on the inorganic plots, yield of G06 was significantly higher (*p* < 0.05) than G14N, E4, and E5 (Supplementary Fig. 1A) and was at par with T511. Yield was highest for G06 and lowest for E4 in both cultivation systems.

#### Shoot mass

In the organic plots, E4 had significantly lower (*p* < 0.05) shoot mass compared to other genotypes that were at par with each other (Supplementary Fig. 2B). In the inorganic plots, G06 had significantly higher (*p* < 0.05) shoot mass compared to E4 and at par with rest of the genotypes for commercial inoculum.

#### Pod number

Pod number in organic plot of E4 was significantly lower (*p* < 0.05) than that of G06 and G14N, while it was at par with E5 and T511 (Supplementary Fig. 2C). In the inorganic plots, G06 had significantly higher (*p* < 0.05) pod number than that of E4, E5, and G14N and was at par with T511.

#### Nodule number

In both organic and inorganic plots, E4 produced no nodules. Among the other genotypes, G06 had the highest nodule number at par with G14N and significantly higher than E5 and T511 (Supplementary Fig. 2D). In the inorganic plots, all four genotypes had similar number of nodules.

#### Root mass

In the organic plots, root mass was significantly lower (*p* < 0.05) in E4 compared to G06, G14N, and E5 and was at par with root mass for T511 (Supplementary Fig. 2E). In the inorganic plots, root mass for G06 was significantly higher (*p* < 0.05) than E4 and T511 while it was at par with G14N and E5.

#### SPAD

In both organic and inorganic plots, E4 showed significantly lowest (*p* < 0.05) SPAD1, SPAD2, and SPAD3 readings among all the genotypes (Supplementary Fig. 2F, 2G, 2H). In organic plots, SPAD1 was significantly higher in G06 which was at par with G14N and T511. In the inorganic plots, SPAD1 was significantly higher in G06 which was at par with G14N and E5. Similar trends were seen for SPAD2 and SPAD3 readings (Supplementary Fig. 2G, Supplementary Fig. 2H).

### Correlation between microbiome composition and yield parameters

**Fig. 6:**
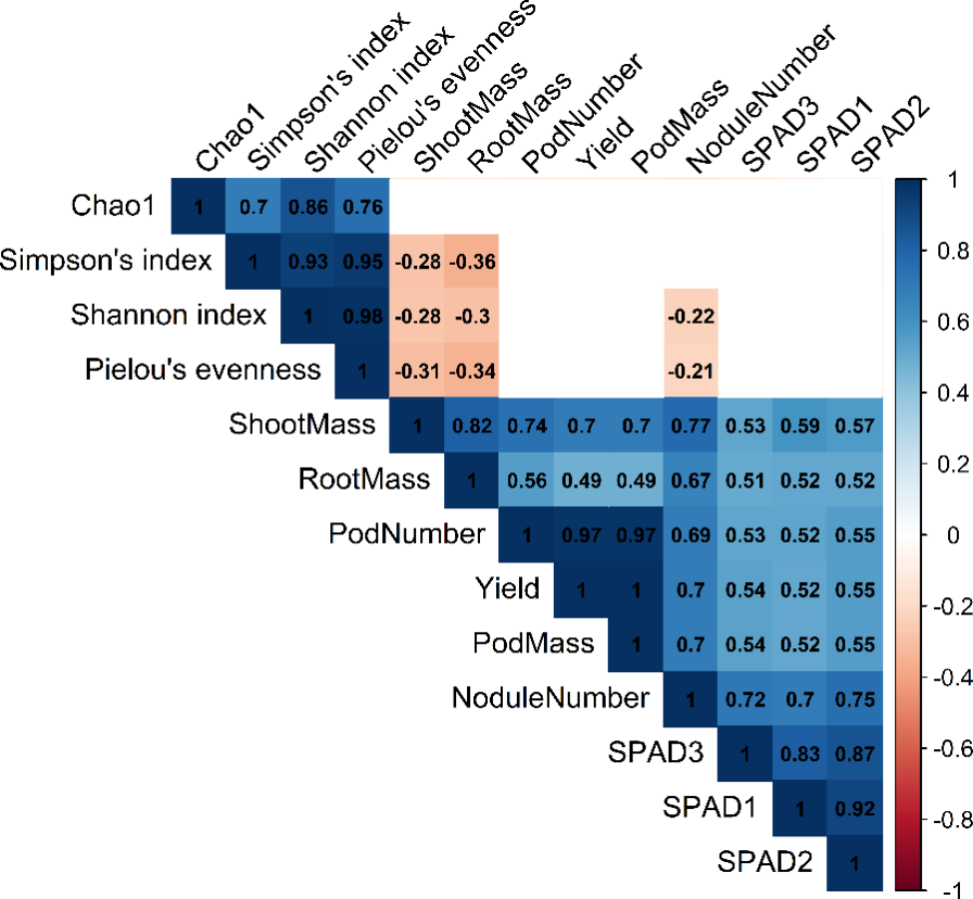
Coefficient of correlation between alpha diversity metrices and yield parameters. Only significant correlations are shown in the figure. Positive correlations are shown in blue color gradient and negative correlations are shown in red color gradient

Significant positive correlations were seen between alpha diversity indices and yield parameters (Fig. 6). Interestingly, significant negative correlations were found for shoot mass and root mass to Simpson’s index, Shannon index, and Pielou’s evenness. There was also a significant negative correlation between nodule number and Shannon index and Pielou’s evenness. However, when the data was independently analyzed for each cultivation type, we did not find any significant negative correlations in the inorganic plot (Supplementary Figure S3). Significant negative correlations in the organic plot existed between root mass and Simpson’s index and Pielou’s evenness (Supplementary Figure S4). Similarly, significant negative correlations were seen between shoot mass and Pielou’s evenness.

## Discussion

Farmers use several farming systems for crop production including conventional system, organic system, bio-organic system, bio-dynamic system, no-till, chisel-till, etc. [24–26]. In this baseline study, we evaluated the impact of organic vs. inorganic cultivation practices in peanuts. These two practices are commonly used, well defined, and have standardized cultivation practices [27, 28]. Analysis of variance performed on alpha diversity indices highlighted a significant effect of genotype, interaction between inoculum and cultivation type, and interaction among all three factors on microbial diversity. Chao1 represents microbial richness, while Shannon index considers both richness and the relative abundance of different groups [29, 30]. Thus, the significantly lower Chao1 and Shannon index under inorganic cultivation system (Fig. 1) suggests that rare microbial species might have disappeared leading to a decrease in evenness of the microbiome composition. Increase in the application on N consistently showed a decreasing trend of bacterial biodiversity measured by Chao1 and Simpson’s index [31]. Therefore, an increase in N deposition in the future might worsen the decline of bacterial diversity. A more diverse bacterial evenness (Simpson’s index) was found in organically managed soils relative to non-organic pastures [32]. Increased diversity of microbial communities in organic soils transforms carbon from organic debris into biomass at lower energy costs and is more efficient in resource utilization [33].

Pielou’s evenness was lower in inorganic plots than that in organic plots showing that inorganic practice had the presence of a dominant species in these samples. Bacterial evenness was found higher in organic pastures compared to non-organic pastures [32]. Organic management might support higher microbiome assemblage [34] and the higher microbial diversity we found in our study is consistent with other studies [33, 35]. Compared to conventional systems, organic systems showed higher biological activity while soil physical and chemical parameters were similar [33]. Restricted use of chemical fertilizers and pesticides in organic fields and application of organic fertilizers might be major factors contributing to higher diversity of microbial communities [36–39].

In the current study, copiotrophic bacteria, such as Proteobacteria, Firmicutes, Gemmatimonadetes, and Bacteroidetes were abundantly present in the soil. These bacteria are found in environments rich in nutrients, particularly carbon [40, 41]. While differences between organic and inorganic practices were seen in the composition of bacteria, it should be noted that the most dominant bacteria were largely the same in both cultivation types with some differences in their relative abundance. This result also suggested that inorganic practice could reduce the abundance of proteobacteria and enhance the abundance of Firmicutes for nodulating legume crops, while organic practice could alleviate the different impacts on bacterial community abundance between Nod+ and Nod- plants. These results are similar to other microbiome studies [32, 42]. Bacterial diversity of Nod+ E5 on organic plots was significantly higher than that in inorganic plots for control treatment indicating that E5 is very responsive towards cultivation practice. The yield of E5 and G14N sustained well in the organic field which suggests that these genotypes could be utilized in organic peanut cultivation with less yield penalty. On the other hand, yield of G06 and T511 were dramatically reduced in organic plots compared to inorganic plots. These genotypes performed excellently in the presence of chemical fertilizers showing that these genotypes are more suitable for conventional instead of organic farming.

Significant interactions were found between cultivation type, inoculum, and genotypes for several yield related traits. A similar trend was seen for most of the yield parameters with highest yield in G06 for both cultivation types. Yields in the organic cultivation system were 10–93% of the yields in the inorganic cultivation system. E4 performed worst in organic practice with yields ranging from 10 to 16% of conventional practice while E5 performed best in organic practice with yields ranging from 67 to 117% of conventional system. Reduction of yields by 60–70% in organic management as compared to conventional management has been found in cereals [43]. Presence of nodules in E5 helped to maintain their yields in organic practice at 67–117% of conventional practice compared to Nod- E4 where the yields were only 10–16% of conventional practice. The presence of weeds in organic fields also decreases the amount of nutrients available for the plants. Interestingly, it has been shown that the yields in organic grasslands were 70–100% of conventional management [33]. Nutrients in organic cultivation are less dissolved in the soil solution and yields in organic systems can be further improved by appropriate plant breeding. Higher levels of fertilization showed that nodule formation and symbiotic N fixation could be reduced by mineral N, while small starter doses of applied N stimulated nodule formation [44]. Meanwhile, the addition of fertilizer treatments increased peanut yield and biomass compared to no fertilization, showing the importance of fertilizer application in peanuts [45].

Significant negative correlations for shoot mass, root mass, and nodule number with some alpha diversity metrices in the combined analysis were observed, which was particularly present in the organic plots. These negative correlations could be due to a decrease in root mass and shoot mass of peanut in organic fields though microbial diversity increased. However, in the inorganic plots, increased root and shoot mass of the peanuts might be due to chemical supplementation of fertilizers that release readily available nutrients to the plants. Studies in other crops have shown that the application of chemical fertilizers contributed heavily towards increasing yield and quality of crops [46, 47].

While several commercial inoculums are available for farmers to plant in the field, we did not find significant changes in the bacterial composition between organic and inorganic plots while using commercial inoculum. Moreover, application of LB8 inoculum on E5 increased the diversity and evenness of bacterial composition. This might be due to preferential affinity of E5 to the LB8 inoculum. Application of this type of microbial inoculant is ideal for a holistic approach to solve agro-environmental problems, as the inoculants support plant growth, nutrient availability and uptake, and support the health of plants [47].

The composition of the plant and soil microbiome is subject to changes on a seasonal basis, and more exhaustive research needs to be done to study the biochemical changes that might occur due to the addition of inoculum to soil. Microbial biomass residues are significant sources of soil organic matter, and their decrease in diversity and biomass may influence the pool size and the chemical composition of soil carbon [48]. Since microbial communities have a central role in ecosystem processes, their decline would impact ecosystem processes and functions [49]. Future studies where a combination of chemical fertilizer and organic fertilizer are applied with novel inoculums could help to find optimum balance between inoculum and external fertilizer application that will increase yield while improving the soil, ultimately contributing to sustainability.

## Materials and methods

### Plant materials

Five peanut accessions were selected for this experiment including three commercial cultivars: Georgia 06G (G06), Georgia 14 N (G14N), and TUFRunner-511 (T511), and two breeding lines, E4 and E5. G06 [50] was selected as this is the most common cultivar of peanut grown in the Southeastern United States and has medium maturity. This cultivar has good yield potential in a wide range of conditions and has a high level of tomato spotted wilt virus (TSWV) resistance [51]. G14N is a runner type peanut and high-oleic, TSWV resistant, and root-knot nematode (RKN) [*Meloidogyne arenaria* (Neal) Chitwood race 1]–resistant [52] and has medium plus maturity. T511 is a cultivar developed by the University of Florida North Florida Research and Education Center (NFREC) and has medium maturity. This cultivar has high oleic oil chemistry, with 76% oleic fatty acid [53]. The cultivars included in this study represented more than 90% of the peanut grown in the Southeastern United States. E4 and E5 are two sister recombinant inbred lines derived from a cross between PI262090 and UF487A, where E4 is a non-nodulating (Nod-) line and E5 is nodulating (Nod+) line [54].

### Experimental design

The field experiment was conducted at the University of Florida Institute of Food and Agricultural Sciences (UF/IFAS), Plant Science Research and Education Unit (PSREU) in Citra, Florida at latitude 29.408780 and longitude -82.144976. The field plot design followed a split-split-plot design with three replications. The farming practice (organic vs. inorganic) was the whole plot. Rhizobia inoculation [commercial inoculum, single-strain inoculum (Lb8), control (blank) inoculum] was the sub-plot. Peanut cultivar (five peanut cultivars) was the sub-sub plot. Each experimental unit was a four-row plot of 4.572 m length x 3.6576 m width containing 30 seeds per row. Peanut field planting, field maintenance, and harvesting were conducted according to the UF/IFAS recommendations for peanut growing in this region. Both organic and inorganic fields were next to each other, but were separated by a traffic road, and were planted on May 2nd of 2019.

### Soil analysis prior to planting

Pre-planting soil samples were collected randomly from three points each in the organic and inorganic plots. Soil samples were submitted to Extension Soil Testing Laboratory (ESTL) at the University of Florida for processing and analysis. Phosphorus and NH_4_N were significantly higher in inorganic plots as compared to the organic plots (Table 2). Therefore, organic fertilizer 10-2-8 at 1120.85 kg/ha was applied prior to planting in the organic plots. There were no significant differences between the other elements.

**Table 2:** Comparison of inorganic and organic cultivation practices and soil analysis of the inorganic and organic plots prior to planting

Factor	Inorganic	Organic
Fertilizer use	Inorganic fertilizer	Organic certified fertilizer
Soil amendments	Gypsum	Organic certified gypsum
Insecticide use	Insecticide	OMRI listed insecticide
Herbicide use	Pre-emergent herbicide, herbicide during cultivation (two times)	Hand-weeding (three times)
Fungicide use	Fungicide	OMRI listed fungicide
**Soil Components**		
P	146.93^a^	119.82^b^
K	16.87	13.38
NH_4_N	0.76^a^	0.53^b^
NO_3_N	1.07	0.88
TKN	261.21	232.6
pH	6.61	6.64

Different letters for soil components indicate significant differences between groups (*p* < 0.05, n = 3)

### Organic cultivation practice

For the organic field, Sulfate of Potash at 56.04 kg/ha were applied at planting. Organic certified gypsum at 2241.70 kg/ha was applied on June 25. Organic certified fertilizer 10-2-8 at 560.42 kg/ha was applied on July 22. Organic Materials Review Institute (OMRI) listed insecticide (Dipel at 1.12 kg/ha) was applied on August 8. Fungicide Double Nickel at 3.36 kg/ha, Serenade at 7 kg/ha, Serenade at 7 kg/ha, basic copper at 1.68 kg/ha, and Serenade at 7 kg/ha were applied on June 7, June 19, July 8, August 6, and August 28, respectively. Hand weeding was done on July 19, August 26, and September 23. The plants were harvested by digging pods from the ground with a digger-shaker-inverter on October 10 (160 days after planting) and left to partially dry in the field for three days. Then, a peanut picker was used to remove the pods from the plant stalk and the pods were dried to 10% moisture level.

### Inorganic cultivation practice

For the inorganic field, preplant fertilizer (5-10-15) at 784.59 kg/ha, fungicide (Abound) at 24oz 1.68 kg/ha in furrow, and insecticide (Imidacloprid) at 0.7 kg/ha in furrow were applied during planting. Pre-emergent herbicides Prowl H20 at 2.33 kg/ha and Strongarm at 0.031 kg/ha were applied on May 3. Dual Magnum herbicide at 1.68 kg/ha was applied on May 13. Fungicide (Bravo at 1.12 kg/ha, Tebustar at 0.5 kg/ha) and insecticide (Dimlin at 0.56 kg/ha) were applied on June 7. Gypsum at 224.17 kg/ha was applied on June 11 followed by herbicide (Cadre at 0.28 kg/ha) on June 12. Fungicide (Bravo at 1.61 kg/ha and Tebustar at 0.5 kg/ha) and insecticide (Dimlin at 0.56 kg/ha) were applied on June 19. Fungicide (Elatus at 0.5 kg/ha and Miravis 0.23 kg/ha) were applied on July 8. Fertilizer 10-10-10 at 560 kg/ha was applied on July 22. Fungicide (Elatus 0.511 kg/ha and Miravis 0.23 kg/ha) were applied on August 6 and (Bravo 1.68 kg/ha and Topsin 0.7 kg/ha) were applied on August 28. The plants were dug on October 10 and followed the same protocol as described above.

### Inoculum

Commercial inoculum (Dyna Start Max) was applied in liquid at the time of planting at the rate of 30 ml per 304.8 m or row according to the manufacturer’s recommendations which was equivalent to 1.04 × 10^11^ viable cells for the entire experiment. The same amount of Lb8 inoculum [23] was prepared and applied in the experiment. For the control, water with no inoculum was used.

### Phenotyping

Plant photosynthetic rate was recorded by SPAD chlorophyll meter readings (SCMR) at 30 (SPAD1), 60 (SPAD2), and 90 (SPAD3) days after planting to monitor peanut leaf chlorophyll content. Three plants from each plot were randomly sampled, and the second fully expanded leaves from the top of the main stems were tested at 09:00–10:00 am at 3 points. After peanut harvesting, total pod yield per plant was measured. Individual nodules were counted for the sample plants. Roots and shoots were separated and measured.

### Soil sampling

Prior to harvest, the peanut roots were removed from the soil with a shovel and then gently shaken to remove the soil that was not tightly attached to the roots. The roots of two plants with tightly attached soil were pooled as one replicate. The rhizosphere soil of sampled roots were collected by placing the specimen in a clean and sterile 50-ml conical tube containing 25 mL of sterile phosphate buffered saline (PBS) solution and vortexing for 15 s [55]. The rhizosphere soil was washed off from the roots and then was poured into a 50-ml Falcon tube, centrifuged and stored at 4 °C for DNA extraction on the same day. This rhizosphere soil sample was also termed as rhizosphere compartment [56, 57].

### DNA extraction and sequencing library preparation

Total genomic DNA was extracted from the rhizosphere soil samples using the DNeasy PowerSoil Pro Kit (QIAGEN) according to the manufacturer’s protocol. Quality and quantity of DNA was checked and sent for normalization, library preparation, and sequencing at CD Genomics, New York, USA. The V4 region of the 16 S rRNA gene was amplified following the Earth Microbiome Project protocol [55]. Sequencing was done using Illumina NovaSeq pair-end 250 bp. Raw sequence data was submitted to the SRA database (BioProject: PRJNA904277).

### 16 S rRNA gene amplification sequence analyses

Reads from 16 S-V4 region were analyzed using Qiime2/v2020.8 [58]. Raw reads were trimmed and dereplicated using DADA2 as implemented in Qiime2 with paired-end setting (including quality control, trimming, pair-joining, and chimera removals). The clean reads were imported into Qiime2 artifacts for data analysis. The 16 S-V4 representative amplicon sequence variants (ASVs) were assigned to the Greengenes database using naϊve Bayes classifier in Qiime2 to produce taxonomy tables.

### Statistical analysis and microbiome characterization

Alpha diversity indexes were calculated for observed sequences, richness, and evenness. Beta diversity analysis included principal coordinate analysis (PCoA) on Bray-Curtis and weighted UniFrac distance. The feature table was rarefied to a sampling depth of 56,000. This sampling depth was selected as it was approaching the maximum depth which retained all samples for our analysis. Alpha diversity metrices were calculated on rarefied OTU tables for comparison groups based on cultivation type, inoculum, and genotype. The α metrics included Chao1, Shannon’s index, Simpson’s index and Pielou’s evenness within each comparison group. Statistically significant differences at adjusted *p*-value < 0.05 were determined using analysis of variance with alpha diversity as the response variable on the y-axis, and cultivation type, inoculum, and genotype as crossed predictor variables on the x-axis. Phenotypic data was analyzed using *agricolae* [59] package in R [60] using split-split plot model. Most of the phenotypes showed significant interaction effects and the data was separately analyzed for organic and inorganic plots. Correlation analysis was done using *corrplot* [61] package in R [60].

## Electronic supplementary material

Below is the link to the electronic supplementary material.

Supplementary Material 1

## Data Availability

Raw sequence data is deposited in the SRA database (BioProject: PRJNA904277).

## References

[1] Gresshoff PM, Ferguson BJ (2017). Molecular signals in nodulation control. Int J Mol Sci.

[2] Fess TL, Benedito VA. Organic versus conventional cropping sustainability: a comparative system analysis. Sustain. 2018;10.

[3] Howieson JG, Dilworth, editors. MJ. Working with rhizobia. Australian Centre for International Agricultural Research: Canberra;; 2016.

[4] Gaskell M, Smith R (2007). Nitrogen sources for organic vegetable crops. Horttechnology.

[5] Velmourougane K. Impact of organic and conventional systems of coffee farming on soil properties and culturable microbial diversity. Scientifica (Cairo). 2016;2016. 10.1155/2016/3604026 PMC4794575 27042378

[6] Seufert V, Ramankutty N. Many shades of gray—the context-dependent performance of organic agriculture. Sci Adv. 2017;3. 10.1126/sciadv.1602638 PMC5362009 28345054

[7] Timsina J (2018). Can organic sources of nutrients increase crop yields to meet global food demand?. Agronomy.

[8] Seufert V, Ramankutty N, Foley JA (2012). Comparing the yields of organic and conventional agriculture. Nature.

[9] Barbieri P, Pellerin S, Seufert V, Smith L, Ramankutty N, Nesme T (2021). Global option space for organic agriculture is delimited by nitrogen availability. Nat Food.

[10] Knapp S, van der Heijden MGA (2018). A global meta-analysis of yield stability in organic and conservation agriculture. Nat Commun.

[11] Lori M, Symnaczik S, Mäder P, De Deyn G, Gattinger A (2017). Organic farming enhances soil microbial abundance and activity—A meta-analysis and meta-regression. PLoS ONE.

[12] Hunter-Cevera JC (1998). The value of microbial diversity. Curr Opin Microbiol.

[13] Vukicevich E, Lowery T, Bowen P, Úrbez-Torres JR, Hart M. Cover crops to increase soil microbial diversity and mitigate decline in perennial agriculture. A review. Agron Sustain Dev. 2016;36.

[14] Hardarson G (1993). Methods for enhancing symbiotic nitrogen fixation. Plant Soil.

[15] Ang BN, Herbert DAJ, Mack TP, Hodges RL (1994). Relationship of pod damage by southern corn rootworm and soil drainage to peanut yield. Peanut Sci.

[16] Singleton PW, Tavares JW (1986). Inoculation response of legumes in relation to the number and effectiveness of indigenous Rhizobium populations. Appl Environ Microbiol.

[17] McDermott TR, Graham PH (1990). Competitive ability and efficiency in nodule formation of strains of *Bradyrhizobium japonicum*. Appl Environ Microbiol.

[18] Badawi FSF, Biomy AMM, Desoky AH (2011). Peanut plant growth and yield as influenced by co-inoculation with *Bradyrhizobium* and some rhizo-microorganisms under sandy loam soil conditions. Ann Agric Sci.

[19] Jeffries P, Gianinazzi S, Perotto S, Turnau K, Barea JM (2003). The contribution of arbuscular mycorrhizal fungi in sustainable maintenance of plant health and soil fertility. Biol Fertil Soils.

[20] El-Shatnawi MKJ, Makhadmeh IM (2001). Ecophysiology of the plant-rhizosphere system. J Agron Crop Sci.

[21] Sessitsch A, Mitter B (2015). 21st century agriculture: integration of plant microbiomes for improved crop production and food security. Microb Biotechnol.

[22] Zhong W, Gu T, Wang W, Zhang B, Lin X, Huang Q (2010). The effects of mineral fertilizer and organic manure on soil microbial community and diversity. Plant Soil.

[23] Paudel DR, Liu F, Wang L, Peng Z, Maya S, Crook M et al. Isolation, characterization, and complete genome sequence of a *Bradyrhizobium* strain Lb8 from nodules of peanut utilizing crack entry infection. Front Microbiol. 2020. 10.3389/fmicb.2020.00093 PMC7020250 32117123

[24] Pfiffner L, Niggli U, Velimirov A, Boltzmann L, Balzer U, Balzer F et al. Effect of three farming systems (bio-dynamic, bio-organic, conventional) on yield and quality of beetroot (Beta vulgaris L. var. esculenta L.) in a seven year crop rotation. In: Workshop on Ecological Aspects of Vegetable Fertilization in Integrated Crop Production in the Field 339. 1992. p. 11–32.

[25] Djurickovic MS. Global warming potential of corn, soybean, and wheat production in organic, chisel till, and no-till farming systems. Hood College; 2010.

[26] Reganold JP, Wachter JM (2016). Organic agriculture in the twenty-first century. Nat Plants.

[27] USDA. National organic program handbook: Guidance and instructions for accredited certifying agents and certified operations. 2011.

[28] Wright D, Tillman B, Small IM, Ferrell JA, DuFault N. Management and cultural practices for peanuts. SS-AGR-74 Agron Dep, Florida Coop Ext Serv, Inst Food Agric Sci. 2016;:10.

[29] Chao A (1987). Estimating the population size for capture-recapture data with unequal catchability. Biometrics.

[30] Shannon CE (1948). A mathematical theory of communication. Bell Syst Tech J.

[31] Wang C, Liu D, Bai E (2018). Decreasing soil microbial diversity is associated with decreasing microbial biomass under nitrogen addition. Soil Biol Biochem.

[32] Acharya M, Ashworth AJ, Yang Y, Burke JM, Lee JA, Acharya RS (2021). Soil microbial diversity in organic and non-organic pasture systems. PeerJ.

[33] Mäder P, Fließbach A, Dubois D, Gunst L, Fried P, Niggli U (2002). Soil fertility and biodiversity in organic farming. Science.

[34] Chen QL, Ding J, Zhu D, Hu HW, Delgado-Baquerizo M, Ma YB (2020). Rare microbial taxa as the major drivers of ecosystem multifunctionality in long-term fertilized soils. Soil Biol Biochem.

[35] Hartmann M, Frey B, Mayer J, Mäder P, Widmer F (2015). Distinct soil microbial diversity under long-term organic and conventional farming. ISME J.

[36] Ashworth AJ, DeBruyn JM, Allen FL, Radosevich M, Owens PR (2017). Microbial community structure is affected by cropping sequences and poultry litter under long-term no-tillage. Soil Biol Biochem.

[37] Yang Y, Ashworth AJ, DeBruyn JM, Willett C, Durso LM, Cook K (2019). Soil bacterial biodiversity is driven by long-term pasture management, poultry litter, and cattle manure inputs. PeerJ.

[38] Sun HY, Deng SP, Raun WR (2004). Bacterial community structure and diversity in a century-old manure-treated agroecosystem. Appl Environ Microbiol.

[39] Chaudhry V, Rehman A, Mishra A, Chauhan PS, Nautiyal CS (2012). Changes in bacterial community structure of agricultural land due to long-term organic and chemical amendments. Microb Ecol.

[40] Fierer N, Lauber CL, Ramirez KS, Zaneveld J, Bradford MA, Knight R (2012). Comparative metagenomic, phylogenetic and physiological analyses of soil microbial communities across nitrogen gradients. ISME J.

[41] Zhang Y, Shen H, He X, Thomas BW, Lupwayi NZ, Hao X (2017). Fertilization shapes bacterial community structure by alteration of soil pH. Front Microbiol.

[42] Lupatini M, Korthals GW, de Hollander M, Janssens TKS, Kuramae EE (2017). Soil microbiome is more heterogeneous in organic than in conventional farming system. Front Microbiol.

[43] Offermann F, Nieberg H. Economic performance of organic farms in Europe. University of Hohenheim, Department of Farm Economics; 2000.

[44] Basu M, Bhadoria PBS, Mahapatra SC (2008). Growth, nitrogen fixation, yield and kernel quality of peanut in response to lime, organic and inorganic fertilizer levels. Bioresour Technol.

[45] Lin X-J, Wang F, Cai H-S, Lin R-B, He C-M, Qing-Hua L (2009). Effects of different organic fertilizers on soil microbial biomass and yield of peanut. Chin J Eco-Agriculture.

[46] Baghdadi A, Halim RA, Ghasemzadeh A, Ramlan MF, Sakimin SZ. Impact of organic and inorganic fertilizers on the yield and quality of silage corn intercropped with soybean. PeerJ. 2018;2018. 10.7717/peerj.5280 PMC6204818 30386686

[47] Adesemoye AO, Kloepper JW (2009). Plant-microbes interactions in enhanced fertilizer-use efficiency. Appl Microbiol Biotechnol.

[48] Simpson MJ, Smith E, Kelleher BP (2007). Microbially derived inputs to soil organic matter: are current estimates too low?. Environ Sci Technol.

[49] van der Heijden MGA, Bardgett RD, van Straalen NM. The unseen majority: soil microbes as drivers of plant diversity and productivity in terrestrial ecosystems. Ecol Lett. 2008;:296–310. 10.1111/j.1461-0248.2007.01139.x 18047587

[50] Branch WD (2007). Registration of ‘Georgia-06G’ peanut. J Plant Regist.

[51] Monfort WS, Prostko EP, Tubbs RS, Harris G, Abney M, Porter WM, et al. UGA peanut production 2020: quick reference guide. Univ Georg Annu Publ AP-118; 2020.

[52] Branch WD, Brenneman TB (2015). Registration of ‘Georgia-14 N’ peanut. J Plant Regist.

[53] Tillman BL, Gorbet DW (2017). Registration of ‘TUFRunner “511” ’ peanut. J Plant Regist.

[54] Peng Z, Tan L, López Y, Maku J, Liu F, Zhou H (2018). Morphological and genetic characterization of non-nodulating peanut recombinant inbred lines. Crop Sci.

[55] Mcpherson MR, Wang P, Marsh EL, Mitchell RB, Schachtman DP. Isolation and analysis of microbial communities in soil, rhizosphere, and roots in perennial grass experiments. 2018; July:1–11. 10.3791/57932 PMC6126543 30102263

[56] Xu J, Zhang Y, Zhang P, Trivedi P, Riera N, Wang Y (2018). The structure and function of the global citrus rhizosphere microbiome. Nat Commun.

[57] Lakshmanan V, Ray P, Craven KD. Rhizosphere sampling protocols for microbiome (16S/18S/ITs rRNA) library preparation and enrichment for the isolation of drought tolerance-promoting microbes. In: Plant Stress Tolerance: Methods and Protocols. 2017. p. 349–62. 10.1007/978-1-4939-7136-7_23 28735410

[58] Bolyen E, Rideout JR, Dillon MR, Bokulich NA, Abnet CC, Al-Ghalith GA (2019). Reproducible, interactive, scalable and extensible microbiome data science using QIIME 2. Nat Biotechnol.

[59] De Mendiburu F. Agricolae: statistical procedures for agricultural research. R Packag. 2014.

[60] R Core Team. R: a language and environment for statistical computing. R Foundation for Statistical Computing; 2021.

[61] Wei T, Simko V, Levy M, Xie Y, Jin Y, Zemla J (2017). Package ‘corrplot ’ Statistician.

